# Structural analogs of 2-(4-fluorophenyl)-6-methyl-3-(pyridin-4-yl)pyrazolo[1,5-a]pyridine for targeting *Candida albicans* non-essential stress kinase Yck2 through protein-ligand binding and dynamics analysis

**DOI:** 10.3389/fchem.2024.1430157

**Published:** 2024-08-13

**Authors:** Ahmed M. Hassan, Aiah M. Khateb, Safaa A. Turkistani, Meshari M. Alhamdan, Raed M. Garout, Vivek Dhar Dwivedi, Esam I. Azhar

**Affiliations:** ^1^ Special Infectious Agents Unit—BSL3, King Fahd Medical Research Center, King Abdulaziz University, Jeddah, Saudi Arabia; ^2^ Department of Clinical Laboratory Sciences, College of Applied Medical Science, Taibah University, Medina, Saudi Arabia; ^3^ Medical Laboratory Sciences Department, Fakeeh College for Medical Sciences, Jeddah, Saudi Arabia; ^4^ Family Medicine Department, Faculty of Medicine, King Abdulaziz University, Jeddah, Saudi Arabia; ^5^ Molecular Diagnostics Laboratory, Clinical Laboratory Department, King Abdulaziz University Hospital, King Abdulaziz University, Jeddah, Saudi Arabia; ^6^ Center for Global Health Research, Saveetha Institute of Medical and Technical Sciences, Saveetha Medical College and Hospitals, Saveetha University, Chennai, India; ^7^ Bioinformatics Research Division, Quanta Calculus, Greater Noida, India; ^8^ Medical Laboratory Sciences Department, Faculty of Applied Medical Sciences, King Abdulaziz University, Jeddah, Saudi Arabia

**Keywords:** yeast casein kinase (Yck2), *C. albicans*, free energy landscape, molecular dynamic simulation, high throughput virtual screening (HTVS)

## Abstract

The rise in drug-resistant fungal infections poses a significant public health concern, necessitating the development of new antifungal therapies. We aimed to address this challenge by targeting a yeast casein kinase of *Candida albicans* for antifungal drug development. The compound library contained 589 chemical structures similar to the previously identified kinase inhibitor GW461484A. Through virtual screening, four compounds with the PubChem IDs 102583821, 12982634, 102487860, and 86260205 were selected based on their binding energies. Hydrophobic bonds and van der Waals interactions stabilised the docked complexes. Comprehensive interaction studies and a 200-nanosecond molecular dynamics simulation suggested that these molecules can maintain stable interactions with the target, as evidenced by satisfactory RMSD and RMSF values. The Rg-RMSD-based Free Energy Landscape of these complexes indicated thermodynamic stability due to the presence of conformers with global minima. These promising findings highlight the potential for developing novel antifungal therapies targeting Yck2 in *C. albicans*. Further experimental validation is required to assess the efficacy of these compounds as antifungal agents. This research provides a significant step towards combating antifungal resistance and opens up a new avenue for drug discovery.

## 1 Introduction

The incidence of invasive fungal infections has increased significantly in recent years due to a higher number of immunocompromised patients with various diseases (Bajpai et al., 2019). These infections, which occur when yeasts or molds invade deep-seated tissues, are severe and often fatal compared to superficial fungal infections (Lamoth et al., 2019). Recent data shows that around 1.9 million people annually face acute invasive fungal infections, and it is estimated that globally, chronic severe fungal infections affect approximately 3 million individuals. Many of these cases result in life-threatening conditions and contribute to more than 1.6 million deaths each year attributed to all fungal diseases (Fang et al., 2023). Additionally, the global rise of multi-drug resistant fungal species is concerning as it complicates treatment and exacerbates mortality rates. Also, the limited options for systemic antifungal treatment pose a significant clinical challenge in managing IFIs (AlMaghrabi et al., 2023). In order to combat these challenges posed by fungal infections, it is essential to develop new classes of antifungal drugs that are effective against drug-resistant strains of fungi and to explore alternative treatment strategies (Mueller et al., 2021).

Candidiasis is a widespread infection that presents significant health challenges, particularly for individuals with weakened immune systems (Pappas et al., 2018). *Candida* species have developed natural and acquired resistance to various antifungal drugs (Sanguinetti et al., 2015). As a common organism in the human skin and gut, *Candida albicans* can cause invasive fungal diseases worldwide due to its ability to change its form in different environments (Talapko et al., 2021). Echinocandins are recommended as an effective treatment for invasive candidiasis because they target the synthesis of the fungal cell wall by specifically inhibiting the glucan synthase enzyme (Perlin, 2015). Prolonged use of antifungal prophylaxis or treatment creates favourable conditions for the development of antifungal resistance among patients (Perlin et al., 2017). The increasing prevalence of multidrug-resistant strains of fungus, particularly in the case of *C. albicans*, poses a significant challenge for the treatment of invasive (Arendrup and Patterson, 2017). This demands, research on new cellular targets within the genomic era and the development of drug-like chemical molecules (Janga and Tzakos, 2009).

A study employing chemical screening to uncover kinase inhibitors identified a fungal version of casein kinase 1, referred to as Yck2 protein, in C. albicans. This protein is deemed an appropriate target for addressing echinocandin resistance due to the morphogenesis of *C. albicans* (Caplan et al., 2020a). The casein kinase 1 family comprises highly preserved serine/threonine kinases found in most eukaryotic organisms (Knippschild et al., 2005). Within *C. albicans*, the Yck2 protein plays a role in nutrient sensing mechanisms, and is an important factor in cell wall regulation; its gene expression significantly increases the enzyme’s activity during interactions with endothelial cells (Childers et al., 2020). Furthermore, the Yck2 protein responds to cell wall stress. Targeting proteins that serve as central hubs controlling stress responses within cellular circuitry may be a compelling approach to weaken fungal infections, enhance traditional antifungal drug efficacy, and address resistance issues (Jung et al., 2017).

These findings suggest that the Yck2 protein is a significant drug target. Recently drug identification via *in silico* approaches has been cost-effective and less time-consuming. Also, this approach provides a piece of detailed information about the dynamic and kinetics of the drug during the inhibition. A recent study identified five potential drug-like compounds exhibiting inhibitory activity against the Yck2 protein of *C. albicans* through various computational techniques (Rabaan et al., 2024). However, the need for inhibitors against this target still prevails. So, our study aims to identify chemical compounds that are structurally similar to the existing inhibitor using computational approaches to overcome the echinocandin resistance developed by the *C. albican’s* morphogenesis.

## 2 Methodology

### 2.1 Target protein retrieval and preparation

The Yck2 kinase protein of *C. albicians* was retrieved from the 3D protein structure database; Protein Data Bank (PBD) (Berman et al., 2000; Caplan et al., 2020a). The identification code for this protein was 6U6A. The retrieved 3D crystal structure weighs around 35.98 kDa and has a resolution of 2.45 Å. The crystal structure was optimised using the UCSF Chimera software before the experiment by adding the required charges and hydrogen to maintain the protonation state (Pettersen et al., 2004). Subsequently, the water molecules and the ions present in the structure were also removed for optimization.

### 2.2 Ligand library preparation

A comprehensive search was conducted within the PubChem database to identify chemical molecules demonstrating 3D structural similarity to the co-crystallized ligand GW461484A (Q0J) (Kim et al., 2019; Caplan et al., 2020a). This search yielded a total of 589 distinct chemical compounds exhibiting such structural resemblance. Subsequently, these compounds were combined to form the ligand library for further virtual screening process.

### 2.3 High throughput virtual screening analysis (HTVS)

HTVS of the ligands with the target protein was performed for the identification and selection of the top-hit chemical molecules. For performing the HTVS MTiOpenscreen webserver was employed (Labbé et al., 2015; MTiOpenScreen, 2024). For the docking of the ligands, a docking grid was built by calculating the coordinates of the binding site of the co-crystalised ligand Q0J. Based on the calculation a docking grid containing centre coordinates of X: 18.67, Y: −14.47, and Z: 14.74 with a size of 20 Å × 20 Å × 20 Å was built and the grid was used for accurate docking of the selected chemical screening process. The selection of the top chemical compounds was based on the scoring function. The molecule with the highest negatively symbolled docking score aka binding energy was selected for further experimental validation.

### 2.4 Molecular re-docking

The validate the binding affinity, the selected chemical molecules were again re-docked with the target protein. For this, the target protein was prepared using the Dockprep tool available in the Chimera software, along with that, each selected chemical molecules were also optimised by adding the appropriate charge and hydrogen atoms to their structures respectively (Pettersen et al., 2004). Further, the re-docking of each chemical compound with the target protein was performed by utilising the previously used docking grid coordinates via the AutoDock Vina plugin available in the Chimera software (Eberhardt et al., 2021). Also, for comparison the co-crystalised ligand Q0J was re-docked using the same parameters. The binding site was validated by superimposing the docked complex over the co-crystallised protein-ligand complex using USCF Chimera software (Pettersen et al., 2004) These generated complexes were visualised using the PyMol and Biovia Discovery Studio Visualizer (DeLano, 2002; Dassault Systemes, 2021). These softwares were used to create the 3D and 2D protein-ligand interaction diagram for the binding affinity analysis, respectively.

### 2.5 Molecular dynamic (MD) simulation

The binding stability and behaviour of the selected protein-chemical molecule complexes in a dynamic environment were analysed using the Amber Molecular Dynamics Package (Case et al., 2023). In this simulation package, the Generalized Amber Force Field (GAFF) is incorporated with the ANTECHAMBER module (Wang et al., 2004; Case et al., 2005). The protein-ligand complexes were solvated and optimised by employing the Tleap module available in the Amber suit. A system was built by placing the complexes in a box containing the TIP3P water module to maintain the aqueous environment around the complexes (Mark and Nilsson, 2001). Next, this system was neutralised by inserting chloride ions to balance the physiological conditions prior to the minimisation of the system. This also helps to remove the steric hindrances or any geometrical disorientation. Further, the system was heated up to 300 K in a progressive pattern, by keeping the atomic pressure to 1 bar with the help of Berendsen barostat (Berendsen et al., 1984). Subsequently, the system was equilibrated and the SHAKE algorithm was used to restrict the hydrogen atoms under constant pressure and temperature (Hess et al., 1997). For maintaining this Langevin thermostat having a collision frequency of 1 ps was used. Finally, the production run of the system was performed at 200 ns (Davidchack et al., 2009). To determine the stability and flexibility of these systems under these conditions, the final simulation trajectory of each complex was extracted and calculated to get their RMSD, RMSF, and Rg values along with the first and last pose interaction analysis.

### 2.6 MM/GBSA-based binding free energy calculation

The binding free energy validates the binding stability and affinity of the protein-ligand complex. For calculating the binding free energy MM/GBSA method was employed by utilising the MMPBSA.py module available in the AMBER package (Miller et al., 2012). For this calculation, the poses generated during the last 50 ns from the 200 ns simulation trajectory of each complex were utilised. The MMGBSA calculation provides the total binding energy along with that the energy disassociation parameters were also calculated.

### 2.7 Rg-RMSD-based free energy landscape (FEL)

FEL analysis offers a thorough insight into the structural dynamics of a molecular complex, facilitating the characterization of its conformational changes and the identification of significant stable states and transition routes. By obtaining the Radius of Gyration (Rg) and Root Mean Square Deviation (RMSD) data throughout the simulation, we generated the FEL plot. Utilizing the Geo-Measure plugin within PyMOL, we visualized the FEL, where each data point represents the energy associated with a specific conformational state distinguished by its Rg and RMSD values (DeLano, 2002; Kagami et al., 2020).

## 3 Results

### 3.1 Virtual screening analysis

**FIGURE 1:**
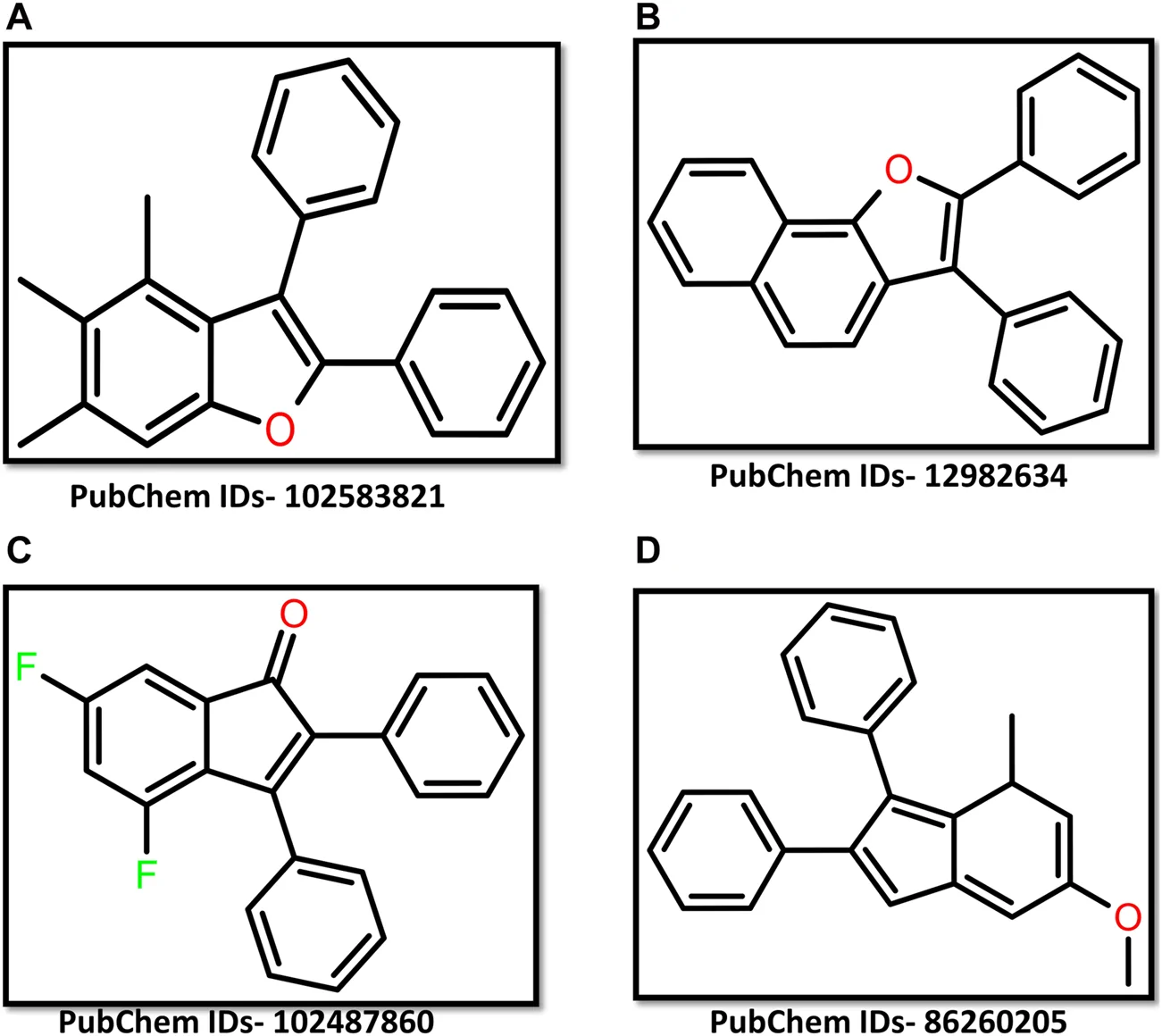
2D representation of ligand **(A)** 102583821 **(B)** 12982634 **(C)** 102487860 **(D)** 86260205.

A total of 589 chemical compounds exhibiting 3D structural similarity were screened. The screening results displayed binding energy from −11.2 kcal/mol to −6.1 kcal/mol (Supplementary Table S1). The chemical molecules were ranked according to the energy obtained and based on energy four chemical molecules having PubChem IDs- 102583821, 12982634, 102487860, and 86260205 were selected. The binding energy of these compounds was −11.2 kcal/mol, −10.9 kcal/mol, −10.9 kcal/mol, and −10.8 kcal/mol respectively. These energy values indicate that these compounds have a strong binding ability with the target protein. For further validation of the binding affinity, the selected chemical compounds were re-docked separately (Figure 1).

### 3.2 Re-docking and binding affinity analysis

**FIGURE 2:**
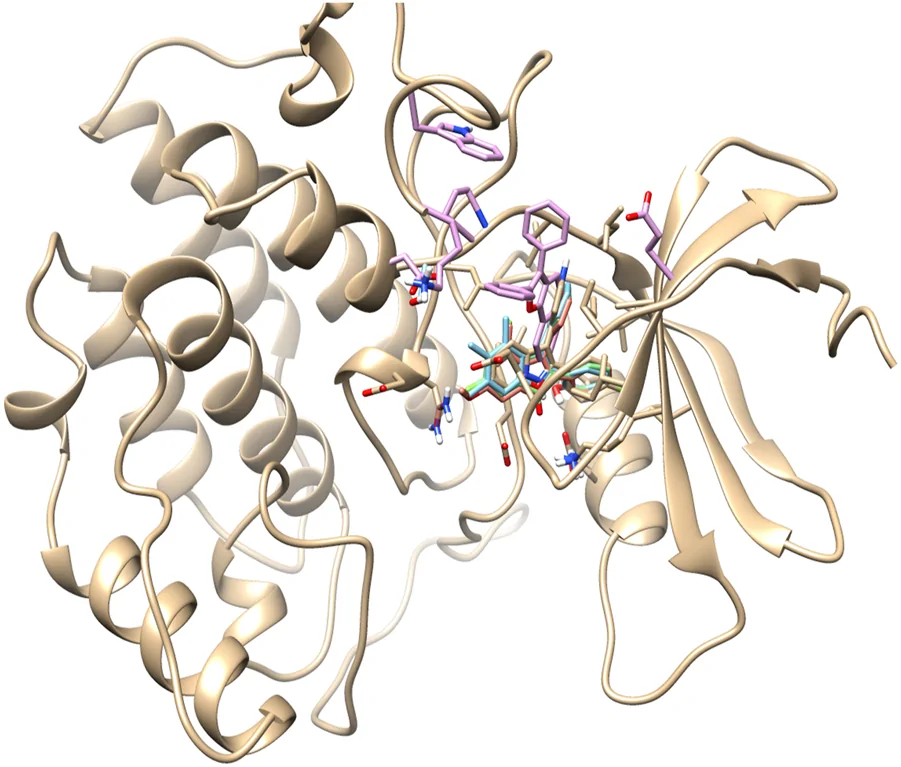
3D representation of superimposition of the docked complex, over the co-crystallised complex.

The re-docking of the selected chemical compounds with the target protein validated the binding energy. The re-docking scores of the Yck2-102583821, Yck2-12982634, Yck2-102487860, and Yck2-86260205 were −11.0 kcal/mol, −10.8 kcal/mol, −10.9 kcal/mol, and −10.7 kcal/mol, respectively. These re-docking scores confirm that no false positive compounds were selected from the obtained screening results, as the re-docking score and the binding energy obtained during the screening were in the same range. For the comparative analysis, the co-crystalised ligand Q0J re-docked with the target protein exhibited a docking score of −10.4 kcal/mol. These findings suggest that the selected chemical compounds showed strong binding affinity with the target protein compared to the native ligand Q0J. Also, the superimposition of the docked complex with the co-crystalised ligand Q0J showed an RMSD value of 0.6 Å, which confirms that the overlapped structures of each docked complex were identical and the ligands are docked in the same binding site (Figure 2).

**FIGURE 3:**
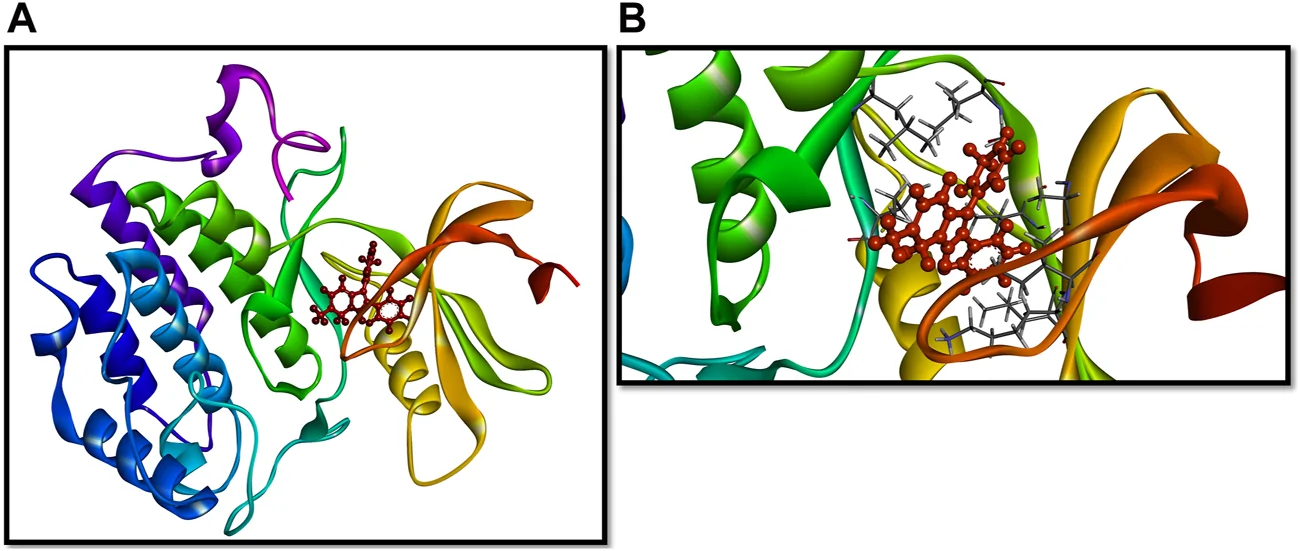
**(A)** 3D representation of the Yck2-Q0J structure and **(B)** binding site of the complex.

Based on the literature analysis, the binding site of the Yck2 protein showed that residues Ile58, Ala71, Lys73, Ile117, and Ile185 are the binding site residues that contribute to hydrophobic bond formation and, Leu120 and Leu115 residues contribute to hydrophobic bond formation. Glu52 residue is present in the loop that encloses the active site and the Ile117 residue is considered as gatekeeper residue along with Ile115. The sequence divergence near the C-terminal end helps in the ligand interaction with the Yck2 protein (Caplan et al., 2020b) (Figure 3)

**FIGURE 4:**
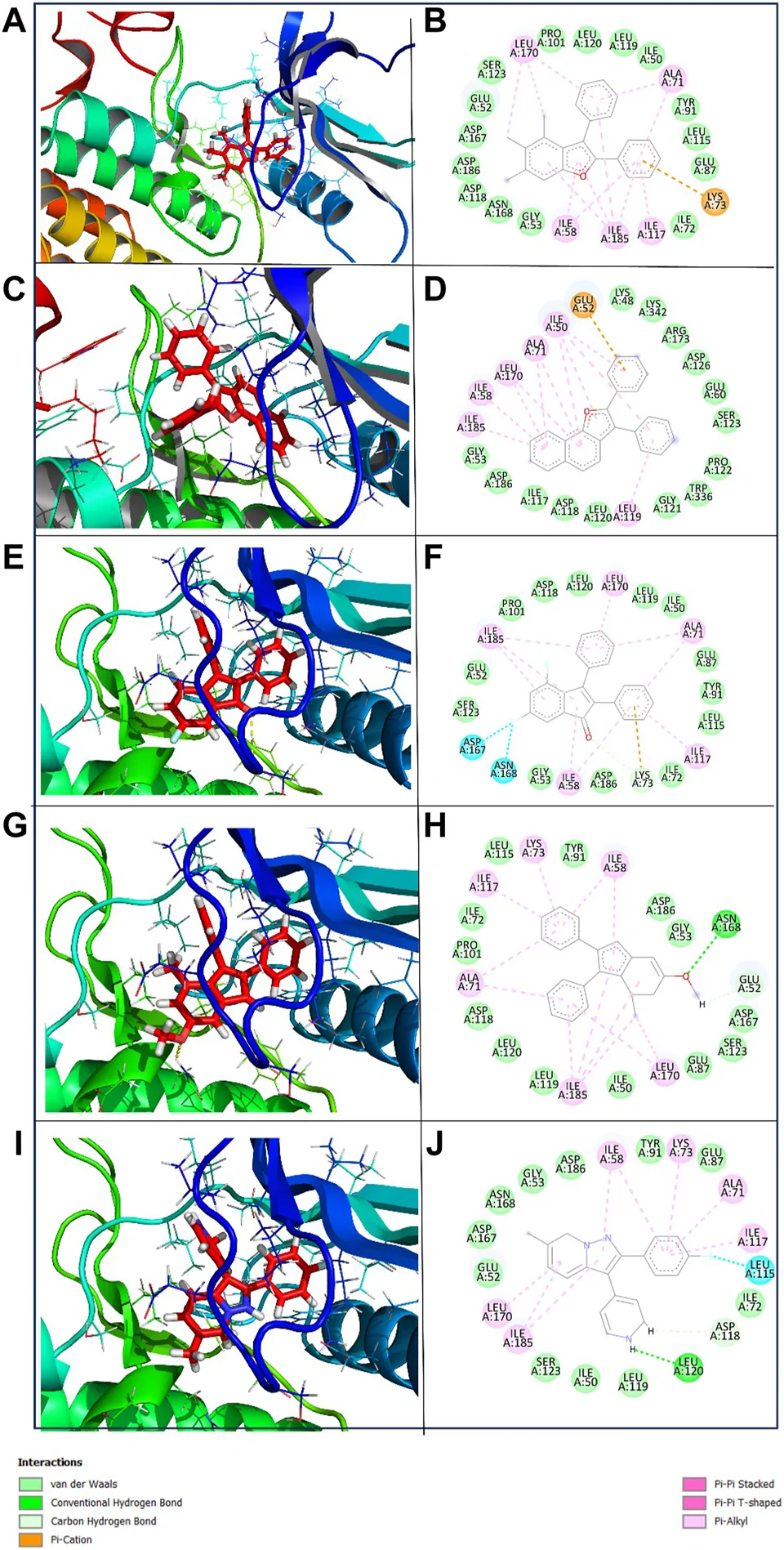
3D and 2D representation of the protein-ligand interaction of the following complexes **(A,B)** Yck2-102583821 **(C,D)** Yck2-12982634 **(E,F)** Yck2-102487860 **(G,H)** Yck2-86260205 and **(I,J)** Yck2-Q0J (reference).

To get a deeper insight into the binding affinity between these selected compounds and the target protein, the intermolecular interactions responsible for forming the binding affinity were studied by creating the 2D interaction diagram of each docked complex and comparing it with the reference complex. According to the 2D plot of the Yck2-102583821 complex, Lys73 residues formed a Pi-cation interaction (4.63 Å) and Ile58 (4.65 Å), Ala71 (5.08 Å), Ile117 (5.20 Å), Leu170 (5.24 Å) and Ile185 (5.14 Å) residues contributed to form Pi-alkyl interaction. Likewise, in Yck2-12982634 complex Glu52 (4.14 Å) residue shows Pi-anion interaction, while Ile50 (4.41 Å), Ile58 (4.80 Å), Ala71 (4.89 Å), Ile117 (5.46 Å), Leu170 (5.26 Å) and Ile185 (5.02 Å) residues were forming Pi-alkyl bonds similar to the first complex. The Yck2-102487860 complex Lys73 (2.88 Å and 4.98 Å) residue participated in both carbon-hydrogen interaction and Pi-cation interaction. In this complex also Ile58, Ala71 (4.78 Å), Ile117 (4.34 Å), Leu170 (5.26 Å) and Ile185 (4.90 Å) residues were forming Pi-alkyl bonds, whereas, Asp167 (3.51 Å) and Asp168 (3.36 Å) interacted with the fluorine atom of the ligand. In the case of the Yck2-86260205 complex Asn168 (2.83 Å) residue established a hydrogen bond with the ligand atom. Also, Glu52 (2.77 Å) formed a carbon-hydrogen bond. Similar to the previous complexes here also Ile58 (4.78 Å), Ala71 (4.48 Å), Ile117 (4.29 Å), Leu170 (5.34 Å) and Ile185 (3.71 Å) contributed to form Pi-alkyl bonds. In the reference complex Yck2-Q0J, Leu120 (2.82 Å) showed hydrogen bond formation, Asp118 (2.63 Å) showed carbon-hydrogen bond, Ile50 (4.42 Å), Ile58 (4.68 Å), Ala71 (4.79 Å), Lys73 (4.23 Å), Ile117 (4.42 Å), Leu170 (5.38 Å) and Ile185 (4.80 Å) formed Pi-alkyl and Leu115 (3.25 Å) interacted with fluorine atom of the ligand. The rest of the active site residues participated in van der Waal interaction with the ligands in all the complexes. The interaction analysis infers that Pi-alkyl interaction contributes to the binding affinity developed between the chemical molecules and the Yck2 Protein (Figure 4; Table 1). Also, these selected chemical compounds showed a similar binding pattern as the reference ligand Q0J. This suggests these compounds may also have the ability to inhibit the target protein same as the native ligand.

**TABLE 1:** The list of residues that participated in major interactions in docked complexes.

S no.	Complex	*H-Bond/C-H bond	Van der Wal	Pi-alkyl	Pi-cation/anion
1	Yck2-102583821	—	Ile50,Glu52,Gly53, Ile72, Glu87, Tyr91, Pro101, Leu115,Asp118, Leu119, Leu120,Ser123, Asp167,Asp168, Asp186	Ile58, Ala71, Ile117, Leu170,Ile185	Lys73
2	Yck2-12982634	—	Lys48, Gly53, Glu60, Ile117,Asp118, Leu120,Ser123, Gly121, Pro122,Arg173, Asp186, Lys342, Trp336	Ile50, Ile58, Ala71, Ile119, Leu170, Ile185	Glu52
3	Yck2-102487860	Lys73	Ile50,Glu52, Ile72, Glu87, Tyr91, Pro101, Leu115,Asp118, Leu119, Leu120,Ser123, Asp186	Ile58, Ala71, Ile117, Leu170, Ile185	Lys73
4	Yck2-86260205	*Asp168, Glu52	Ile50,Gly53, Ile72, Glu87, Tyr91, Pro101, Leu115,Asp118, Leu119, Leu120,Ser123, Asp167,Asp186	Ile58, Ala71, Lys73, Ile117, Leu170, Ile185	—
5	Yck2-Q0J (reference)	*Leu120, Asp118	Ile50, Glu52, Gly53, Ile72, Glu87, Tyr91, Leu119,Ser123, Asp167, Asp168, Asp186	Ile58, Ala71, Lys73, Ile117, Leu170, Ile185	—

### 3.3 Molecular dynamics (MD) simulation

The docking study provided significant evidence about the binding stability of the docked Yck2-chemical molecule complex. However, their stability and flexibility under a solvent-based dynamic environment provided much validation. So, these complexes were simulated for 200 ns under a predesigned solvent-based environment. This provided adequate information about the dynamic stability and flexibility of the complexes. These are obtained by calculating the root mean square deviation (RMSD), and root mean square fluctuation (RMSF) from the simulation trajectory of each complex. Moreover, from the simulation trajectory, the ligand compactness by calculating the radius of gyration (Rg) values was studied.

#### 3.3.1 RMSD calculation

The RMSD evaluation of the protein-ligand complex is a key metric for studying the conformational dynamics and flexibility of the complexes. This helps to understand how the binding of the ligands influences the protein structure and dynamic nature. Based on the RMSD plot analysis of all the selected Yck2-chemical compound complexes, the Yck2 protein in all the complexes was stable throughout the simulation and did not endure any structural conformational changes due to the binding of the selected chemical molecules. Except for the protein in the Yck2-102487860, the protein RMSD value in all the complexes was less than 3 Å. In the case of the Yck2-102487860 complex, the RMSD value till 180 ns was less than 3 Å, but during the last 20 ns an insignificant rise up to 3.5 Å was observed. However, this change does not affect the overall stability of the protein during the simulation. During the analysis of the reference complex, the Protein RMSD was 2 Å. This confirms that similar to the native ligand, the binding of the selected chemical molecules also stabilises the protein, without any conformational changes.

**FIGURE 5:**
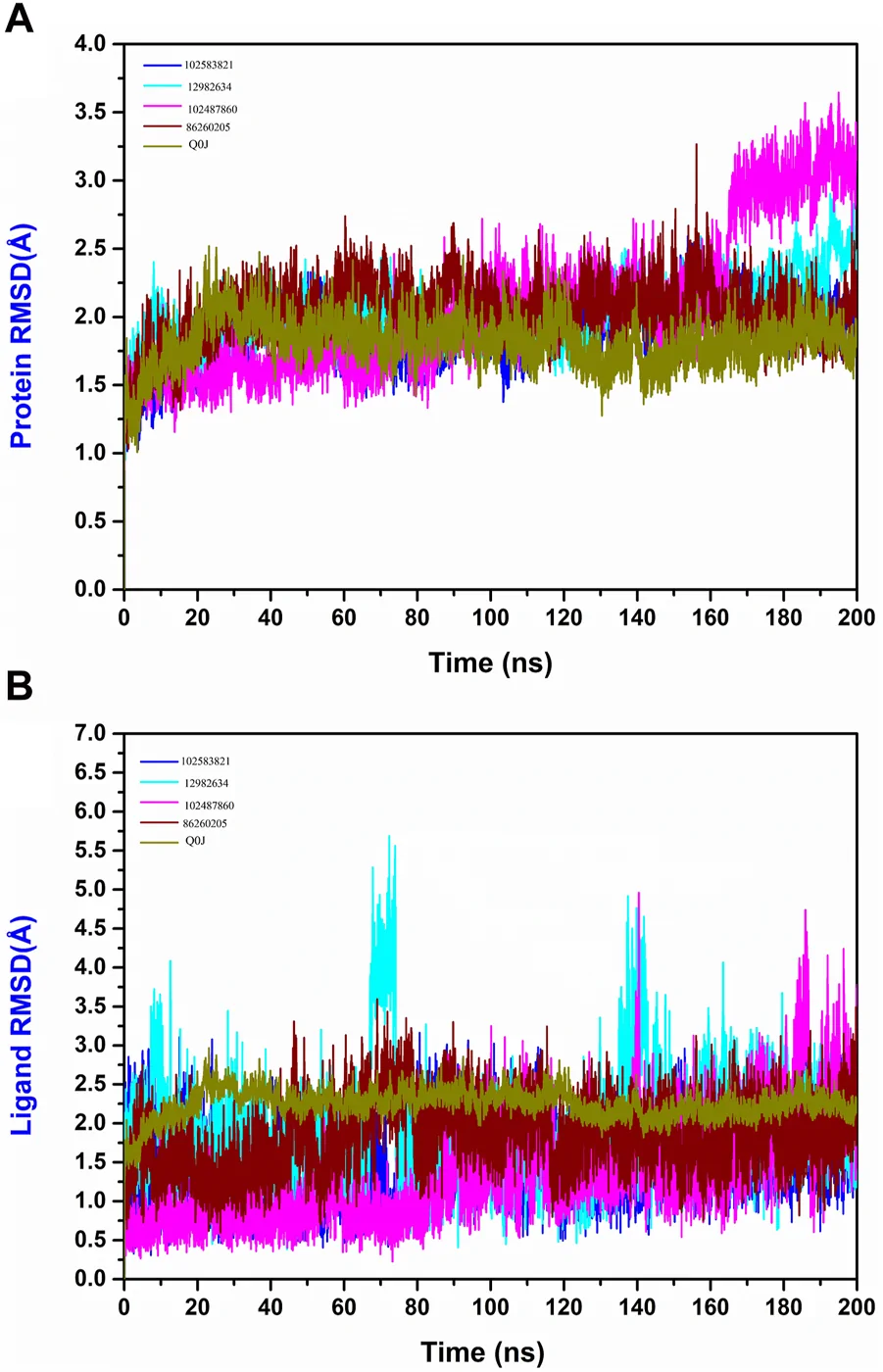
**(A)** Protein RMSD and **(B)** Ligand RMSD plot generated from the 200 ns MD simulation trajectory of the following complexes Yck2-102583821, Yck2-12982634, Yck2-102487860, Yck2-86260205 and Yck2-Q0J (reference).

Furthermore, the Ligand RMSD of each complex were also analysed. The ligand 102583821 showed maximum stability with an RMSD value of less than 3 Å. Similarly, the ligand 12982634 showed an overall stable nature during the simulation. The ligand exhibited significant deviations between 60 and 80 ns (5.5 Å) and 140–150 ns (4.5 Å). However, these deviations did not contribute to structural deviation or ligand instability during the simulation. The chemical molecule 102487860 also displayed stable behaviour during the simulation. From the beginning of the simulation till 90 ns the RMSD value was less than 2 Å. After 90 ns the RMSD value increased to 3 Å, at 140 ns a major deviation reaching up to 5 Å was observed, however, that remained for a short period and the value was reduced to 3 Å and by the end of the simulation deviations till 4 Å were again observed, which gradually declined to 3 Å in the last 10 ns of the total simulation time. This suggests that the observed deviations could have led to the disruption of pre-existing interactions or the formation of new interactions within the protein-ligand complex. However, these deviations do not lead to any conformational changes and the ligand remains bound to the protein in a stable state. The 86260205 chemical compounds also showed maximum dynamic stability and exhibited an RMSD value of 3 Å. The native ligand Q0J’s RMSD value was 2.5 Å. These observations confirm that the identified ligands are dynamically stable. Compared to the reference complex, the selected complexes show the presence of deviations at various times of simulation, but these deviations do not affect the stability of the complexes. These findings validate that these compounds do have similar traits to the reference compound and may have the ability to inhibit the target protein in a similar pattern (Figure 5).

#### 3.3.2 RMSF calculation

**FIGURE 6:**
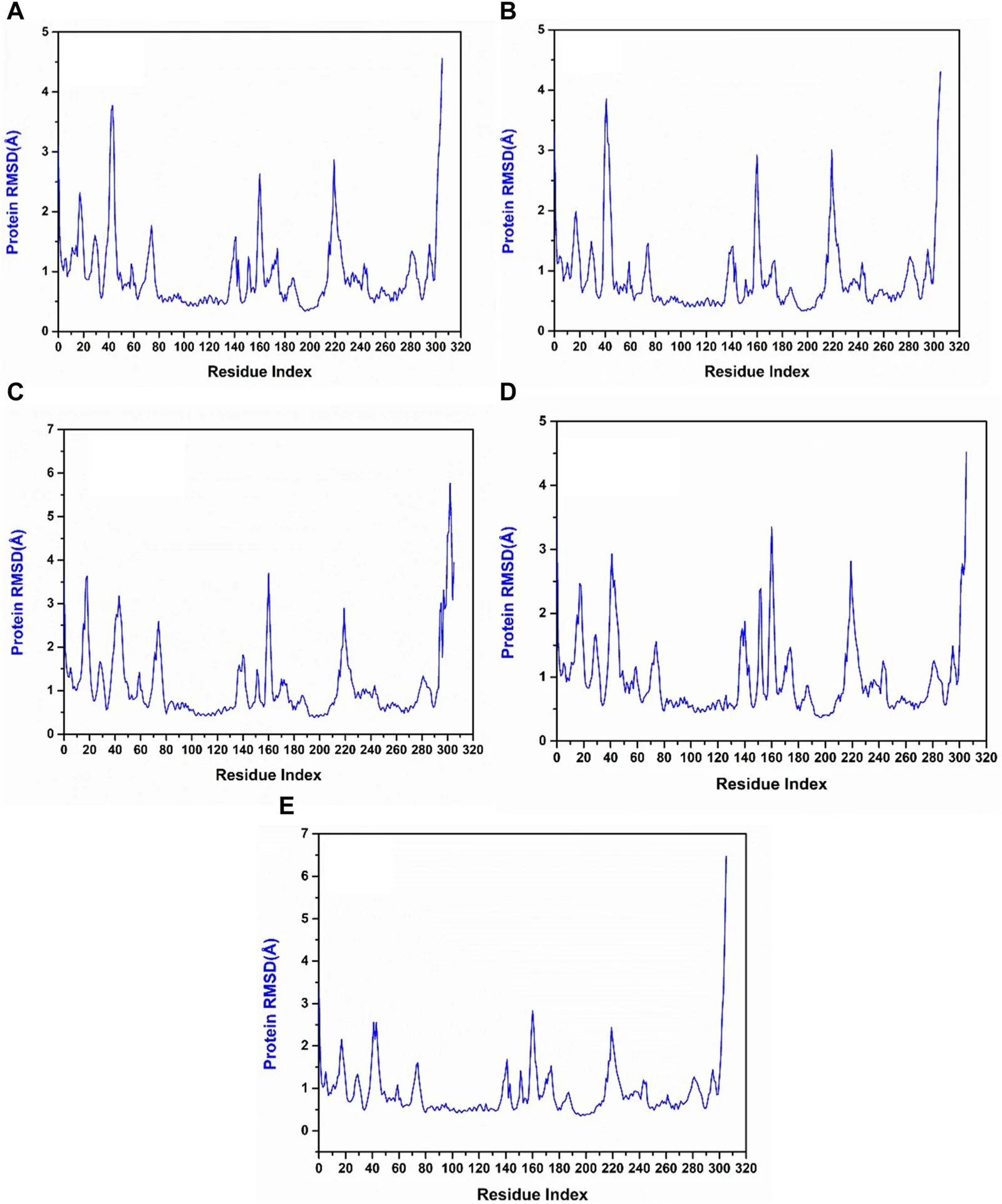
RMSF plot generated from the 200 ns MD simulation trajectory of the following complexes. **(A)** Yck2-102583821, **(B)** Yck2-12982634, **(C)** Yck2-102487860, **(D)** Yck2-86260205 and **(E) **Yck2-Q0J (reference).

The RMSF of protein measures the residual flexibility during the simulation. It helps to identify the mobile region of the protein structure. In the protein RMSF plot of all the simulated complexes, the residues between 40 and 60 showed fluctuations up to 3.5 Å, whereas the residues at 140–160 and 210–230 exhibited fluctuations up to 3 Å. In the reference complex’s RMSF plot also these residues were showing fluctuations. These observations infer that these regions are the flexible and most mobile regions of the protein structure. However, these fluctuations were not responsible for any structural changes and the protein remained in a stable state (Figure 6).

#### 3.3.3 Initial and final pose analysis

**FIGURE 7:**
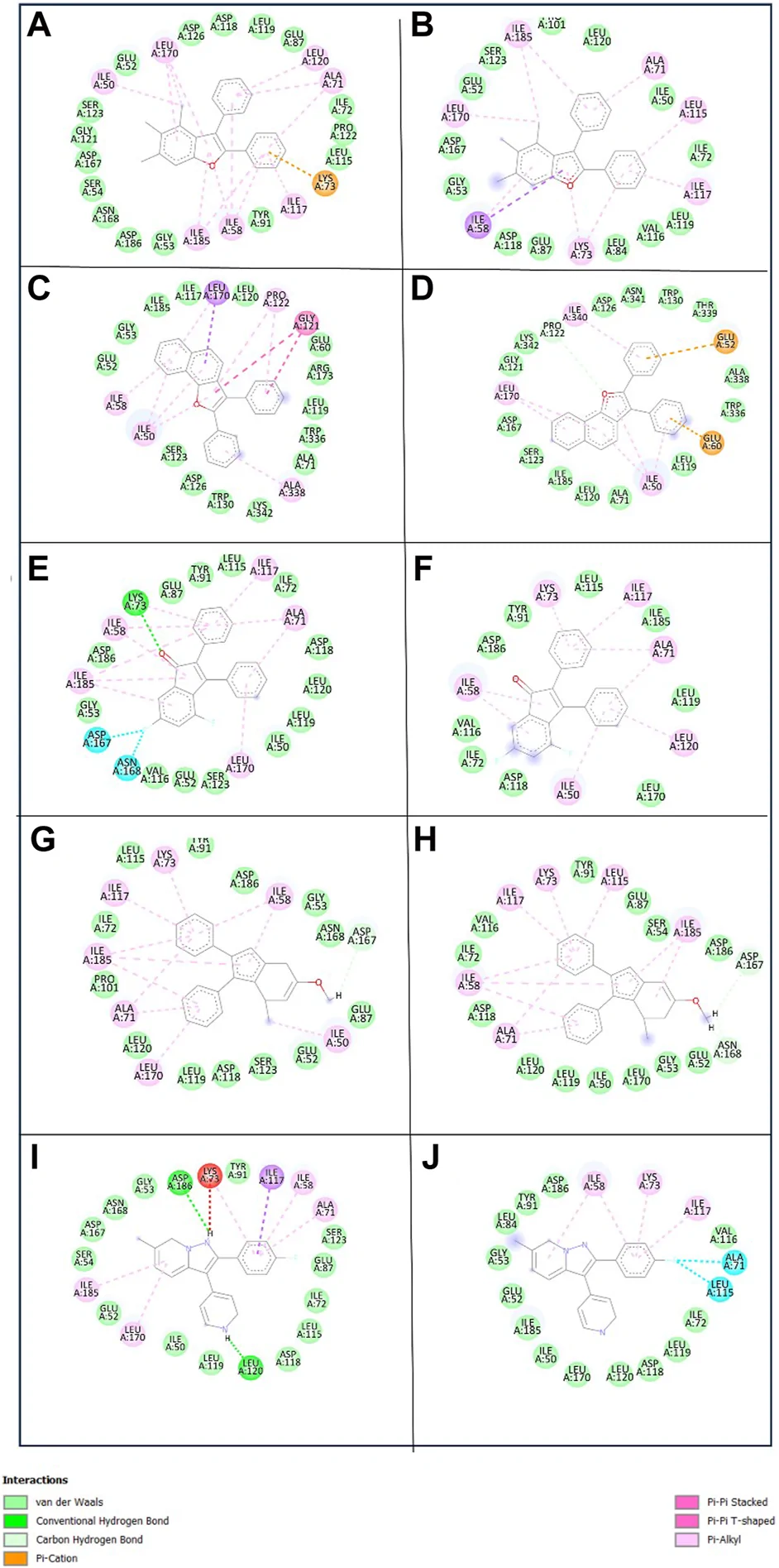
Initial and final pose interaction of the following complexes **(A,B)** Yck2-102583821 **(C,D)** Yck2-12982634 **(E,F)** Yck2-102487860 **(G,H)** Yck2-86260205 and **(I,J)** Yck2-Q0J (reference) extracted from 200 ns simulation trajectory.

The interaction pattern changes during the simulation. These are crucial for understanding the binding stability of the complexes during the simulation process. For this analysis, the initial pose and the final pose of each complex were extracted. Subsequently, 2D interaction diagrams of these poses were generated to analyse the difference in the binding pattern. Based on the 2D interaction diagram the initial pose of the Yck2-102583821 complex Ile58 residue forms a Pi-alkyl bond, whereas by the end of the simulation, this residue also forms a Pi-sigma bond with the ligand atoms. In the Yck2-12982634 complex, Pro122 forms a Pi-alkyl bond, but in the last pose, it forms a carbon-hydrogen bond. Further, Gly121 residue displayed amide-pi stacked interaction, but by the end, it formed van der Waal interaction. Glu52 and Gly60 displayed van der Waal interaction, however, it exhibited Pi-cation interaction by the end of the simulation. Likewise, in the Yck2-102487860 complex, the initial pose displayed a hydrogen bond with Lys73 and, Asp167 and Asp168 residues interact with the fluorine atom of the ligand. But, in the last pose these interactions were absent only van der Waal and Pi-alkyl interactions were formed between residues and ligand atoms. In the case of the Yck2-86260205 complex, only Asp167 forms a carbon-hydrogen bond, but in the last pose along with Asp167, Asp168 also forms a carbon-hydrogen bond. Moreover, in the case of the reference complex, the Ile117 residue forms a Pi-sigma bond and has no interaction with the fluorine atom in the initial pose. In the last pose, Ile117 residue participates in Pi-alkyl and, Ala71 and Leu115 residue interact with the fluorine atoms. These findings suggest that during the simulation certain residues form new bonds to stabilise the complex (Figure 7).

#### 3.3.4 Radius of gyration (Rg) determination

**FIGURE 8:**
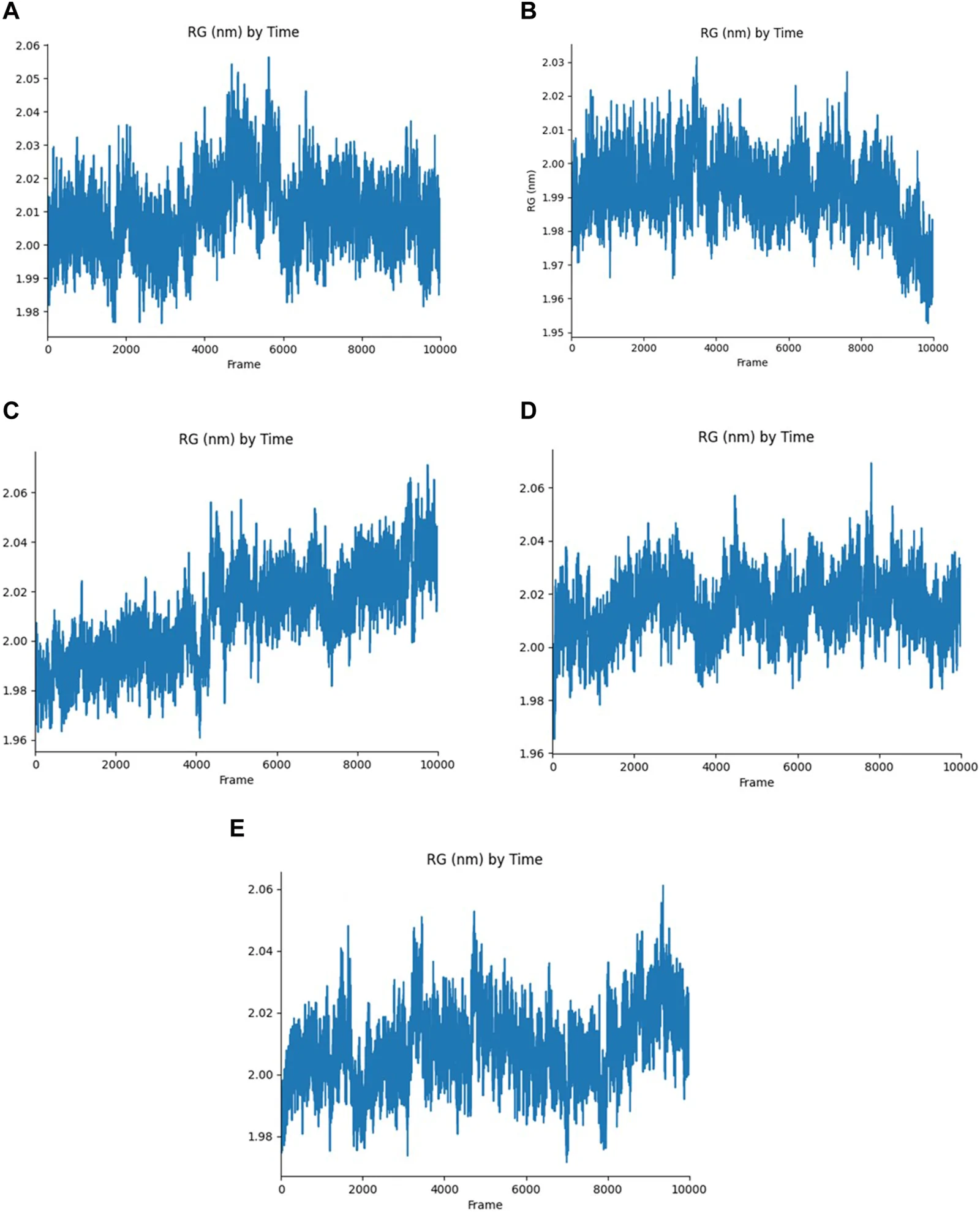
Rg plot generated from the 200 ns MD simulation trajectory of the following complexes **(A)** Yck2-102583821 **(B)** Yck2-12982634 **(C)** Yck2-102487860 **(D)** Yck2-86260205 and **(E)** Yck2-Q0J (reference).

The Rg of the protein-ligand complex measures the compactness of the structure, It provides an overview of the binding pattern of the ligand and their role in the protein’s conformation. According to the Rg plot, Yck2-102583821 complex fluctuations were observed between 4,000 and 6,000 frames and the fluctuation reaches up to 2.06 nm. In the Yck2-12982634 complex, the Rg values reach up to 2.03 nm, however, a fall in the Rg value (1.99 nm) was observed by the end of the simulation. Interestingly, in the Yck2-102487860 complex, a rise in the Rg value was observed after 4,000 frames. The Rg value was more than 2.06 nm by the end of the simulation. In the Yck2-86260205 complex, fluctuations were observed but the overall Rg value was 2.04 nm. The Rg plot of the reference complex also showed fluctuations that reached 2.06 nm at different time frames. These observations suggest that all the selected structures are significantly compact and show similar compactness as seen in the reference complex. This also suggests that no conformational changes were noticed in the protein-ligand complex (Figure 8).

### 3.4 MM/GBSA-based binding free energy calculation

**FIGURE 9:**
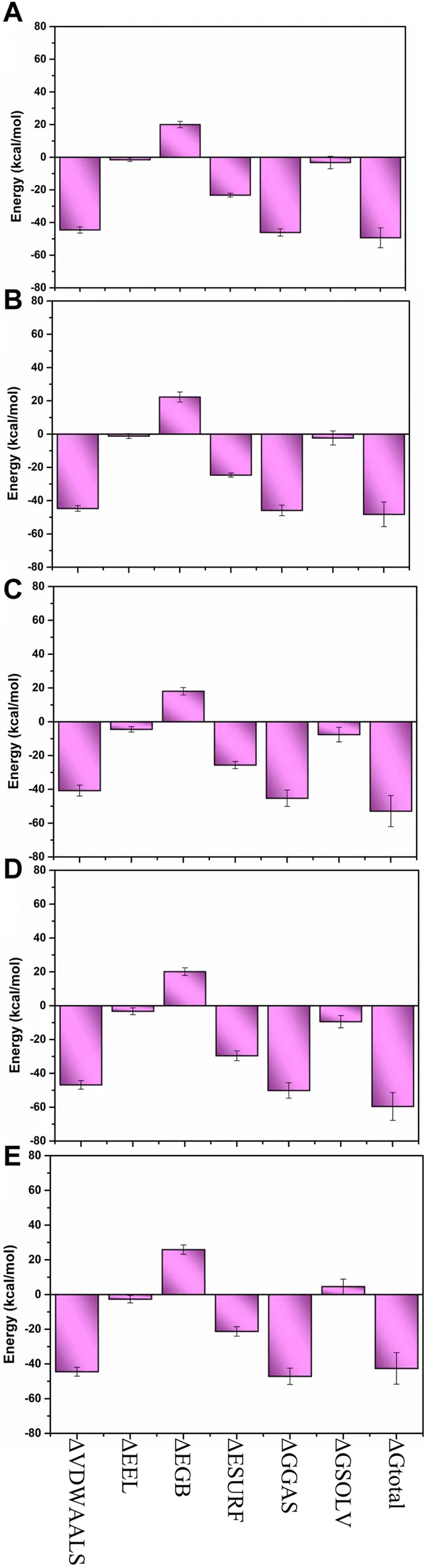
Bar graph representation of MM/GBSA-based energy disassociation components of **(A)** Yck2-102583821 **(B)** Yck2-12982634 **(C)** Yck2-102487860 **(D)** Yck2-86260205 and **(E)** Yck2-Q0J (reference).

The MM/GBSA method was utilized to compute the binding free energy of the Yck2-chemical molecule complexes. This approach determines the necessary binding stability by calculating the complete interaction energy involved in each pose of the complex during the simulation and then averaging these values to obtain the overall free binding energy of the complex. The results indicated that compared to the reference complex (−42.59 kcal/mol) and other selected complexes, Yck2-86260205 (−59.57 kcal/mol) exhibited maximum ΔG_total_ value, demonstrating a high binding affinity with the target protein. Subsequently, Yck2-102487860 (−52.87 kcal/mol), Yck2-102583821 (−49.35 kcal/mol), and Yck2-12982634 (−48.23 kcal/mol) followed in decreasing order of ΔG_total_ values for their respective complexes. Furthermore, an assessment of participating components’ contribution to energy degradation revealed that van der Waals energy and net gas phase energy play significant roles in contributing to binding stability within each complex (Table 2; Figure 9).

**TABLE 2:** The table of the binding free energy calculated along with the energies of energy degrading components.

Energy disassociation components	Yck2-102583821	Yck2-12982634	Yck2-102487860	Yck2-86260205	Yck2-Q0J
Van der Waal energy (ΔVDWAALS)	−44.54 ± 1.91	−44.69 ± 1.68	−40.77 ± 3.23	−46.81 ± 2.53	−44.47 ± 2.55
Electrostatic energy (ΔEEL)	−1.54 ± 1.06	−1.17 ± 1.44	−4.50 ± 1.60	−3.31 ± 2.03	−2.67 ± 2.17
Polar solvation energy (ΔEGB)	20.00 ± 1.92	22.28 ± 3.02	18.03 ± 2.20	20.11 ± 2.28	25.83 ± 2.70
Non-polar solvation energy (ΔESURF)	−23.26 ± 1.17	−24.64 ± 1.21	−25.63 ± 2.14	−29.56 ± 2.87	−21.28 ± 2.70
Net gas phase energy (ΔGGAS)	−46.08 ± 2.24	−45.87 ± 3.13	−45.28 ± 4.83	−50.12 ± 4.56	−47.14 ± 4.72
Net solvation energy (ΔGSOLV)	−3.26 ± 3.83	−2.35 ± 4.24	−7.59 ± 4.35	−9.45 ± 3.65	4.55 ± 4.34
ΔG_total_	−49.35 ± 6.08	−48.23 ± 7.37	−52.87 ± 9.19	−59.57 ± 8.22	−42.59 ± 9.07

### 3.5 Rg-RMSD-based free energy landscape

**FIGURE 10:**
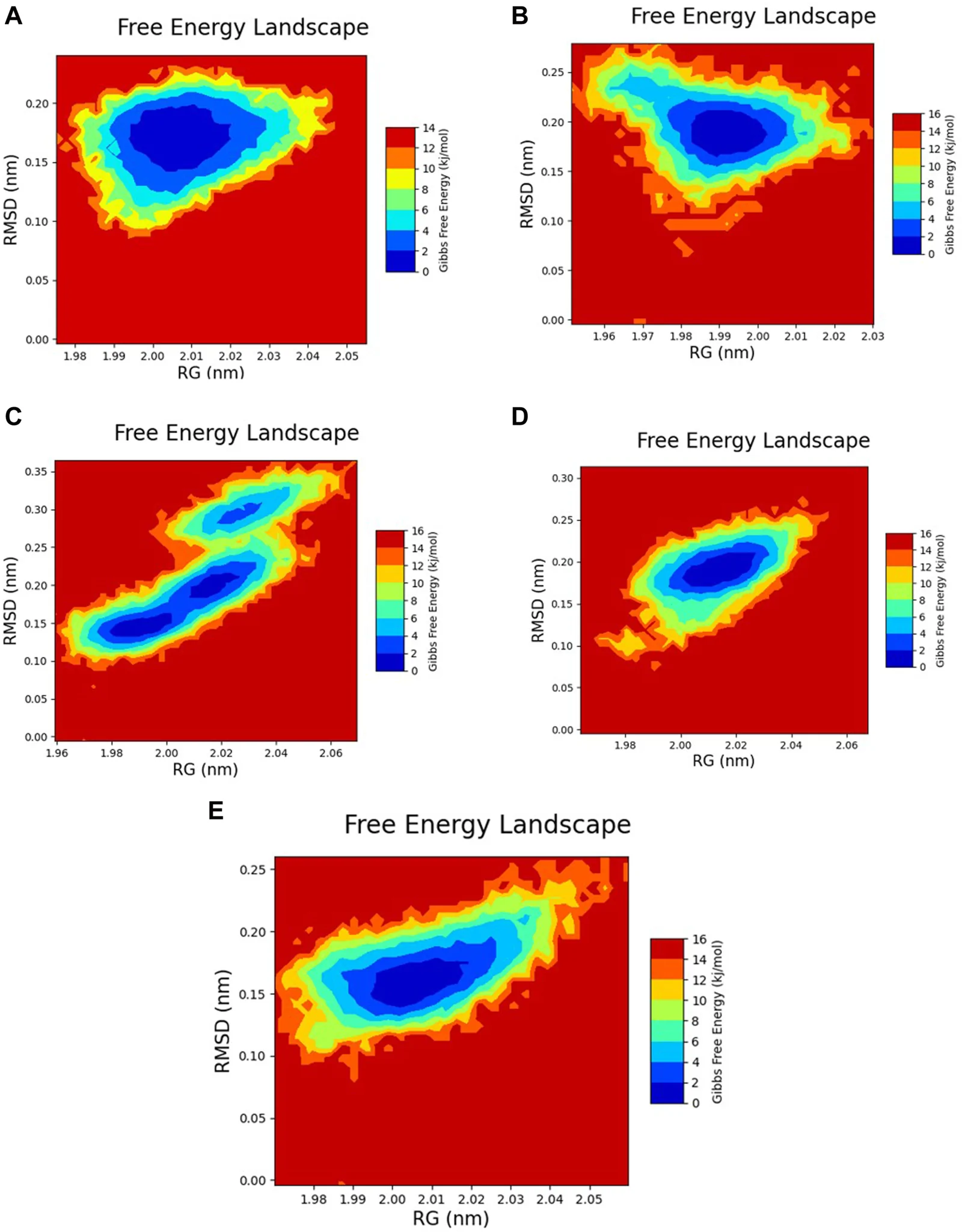
2D FEL plot of the following complexes **(A)** Yck2-102583821 **(B)** Yck2-12982634 **(C)** Yck2-102487860 **(D)** Yck2-86260205 and **(E)** Yck2-Q0J (reference).

The free energy landscape of each complex was graphed to assess their dynamic properties and thermodynamic equilibrium. In this analysis, the rGyr and RMSD values were utilized to create the graphs. The 2D plots revealed that the most stable conformations with global minima exhibited Gibbs free energy within the range of 0–2 kJ/mol. Conformers falling within this range demonstrated maximum stability and structural similarity. Based on the color gradient analysis of the FEL plot, all selected docked complexes except for Yck2-102487860 exhibited maximum thermodynamic stability characterized by a single large basin (dark blue colour), while Yck2-102487860 displayed scattered basins indicating conformational changes in its protein structure. The reference complex also showed dynamic stability due to a single basin, suggesting that all other complexes are stable owing to structurally similar and dynamically stable conformers present in their respective basins. This analysis provides evidence for the conformational stability of the Yck2-chemical complexes and their thermodynamic equilibrium (Figure 10; Supplementary Figure S1).

**FIGURE 11:**
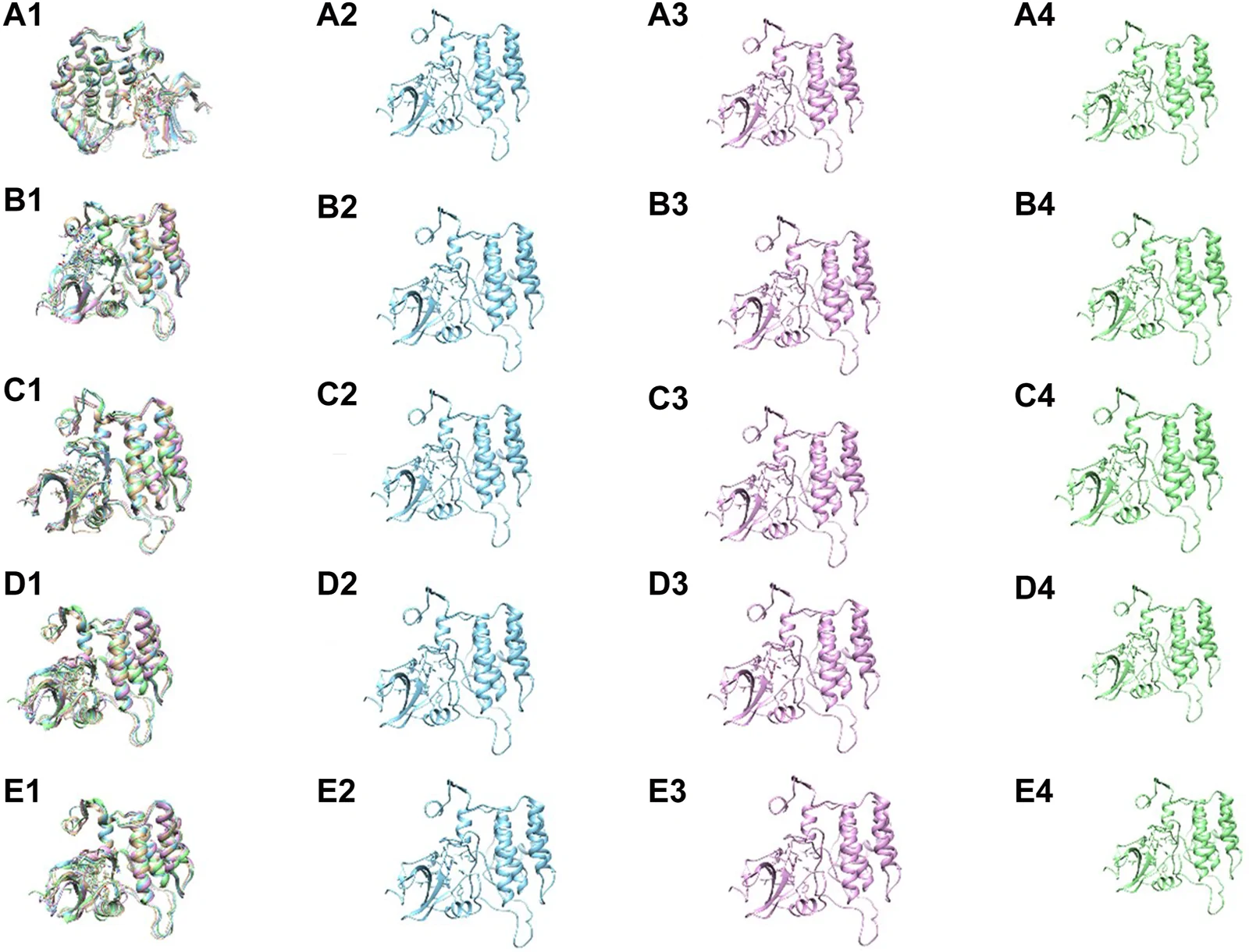
The superimposition and the conformers within the basin of the following complexes **(A**_**1–4**_ **)** Yck2-102583821 **(B**_**1–4**_ **)** Yck2-12982634 **(C**_**1–4**_ **)** Yck2-102487860 and **(D**_**1–4**_ **)** Yck2-86260205 and **(E**_**1–4**_ **)** Yck2-Q0J (reference).

To gain further insight into the conformational stability of the Yck2-chemical molecule complexes, global minima conformers from each basin were extracted and superimposed using 0 kJ/mol Gibbs free energy as a reference point for selecting the most stable conformation during superimposition. Superimposing these structures along with those from other complexes resulted in an overall RMSD value below 1.5 Å, confirming alignment without significant positional displacement and validating their stability at minimum energy state (Figure 11).

## 4 Discussion

Fungal infections significantly contribute to global infectious disease-related mortality. *Candida* species, particularly *C. albicans*, are a frequent cause of invasive fungal diseases (Shariati et al., 2020). Due to the eukaryotic nature of fungi similar to their human host, there is a restricted number of distinct molecular targets available for antifungal research and development (Nicola et al., 2019). Yeast casein kinase (Yck2) plays a crucial role in cell wall regulation and has been identified as a promising drug target for combating drug resistance in *C. albicans*. In this work, we focus on targeting this protein using chemical compounds that are structurally similar to the previously identified kinase inhibitor GW461484A. For this investigation, we have utilised computational drug discovery approaches. Previously, Rabaan and his team used the same approach to target the Yck2 protein of *C. albicans* (Rabaan et al., 2024). We have collected a total of 589 chemical compounds which are structurally similar to the kinase inhibitor from the PubChem database. The compounds were screened via a high throughput virtual screening process and based on the binding energy of four chemical compounds (102583821, 12982634, 102487860, and 86260205) were selected (Wall and Lopez-Ribot, 2020). The highlight of these compounds is that due to their structural similarity with the previously identified kinase inhibitors, they can be utilized as analogues of these known inhibitors. This suggests that these newly identified compounds may have the potential to inhibit the target protein, the yeast casein kinase (Yck2) of *C. albicans*, in a similar manner as the previously studied inhibitors. Further, the binding pattern analysis through molecular interactions showed that these compounds exhibited interactions similar to the known kinase inhibitors, indicating their potential to bind to the target protein effectively. The binding affinity of these compounds was found to be due to the presence of various hydrophobic interactions along with van der Waals forces, which are known to contribute significantly to the stability of protein-ligand complexes. Moreover, these compounds also displayed exceptional dynamic stability under a solvent-based environment, as confirmed by the RMSD plot evaluation, which showed RMSD values around 3 Å. The RMSF plot also revealed common residues that are flexible, providing insights into the dynamic behavior of the protein-ligand complexes. The first and last pose interaction patterns further revealed that hydrophobic bonds contribute to the binding stability of these compounds. The Rg value showed the compactness of the ligand when bound to the protein, indicating a favourable binding mode. The FEL plot based on Rg and RMSD values showed significant thermodynamic stability, excluding the Yck2-102487860 complex. All other complexes, including the reference complex, showed a single basin, confirming that these compounds are dynamically stable. Comparing these findings with the previous study by Rabaan and his team, who identified five drug-like compounds from a diverse-lib database, the current study suggests that the newly identified compounds may have a better potential to inhibit the Yck2 protein. This is because the RMSD plot analysis in the previous study showed that the binding of their identified compounds led to structural deviations in the protein structure, whereas the compounds in the current study did not cause any such conformational changes. Additionally, the previous study used PCA-based FEL plots, which showed dynamic instability, a scenario that was not observed in the FEL plots of the compounds in the current study. These observations collectively suggest that the newly identified compounds may have a better potential to inhibit the Yck2 protein, which could be a significant breakthrough in the field of antifungal drug discovery.

## 5 Conclusion

In conclusion, the utilization of *in silico* methods to discover potential drug candidates against yeast casein kinase in *C. albicans* has proven to be an original and effective strategy. The novelty of targeting Yck2 as an antifungal drug target has been underscored by the identification of promising inhibitors through virtual screening. The compounds 102583821, 12982634, 102487860, and 86260205 have demonstrated strong binding affinity to the target protein, with hydrophobic bonds and van der Waal interaction contributing to this stability. The dynamic stability of these complexes was supported by 200 ns molecular dynamics simulations, which revealed stable RMSD and RMSF values below 3 Å. The free binding energy calculation using the MM/GBSA method validated that these compounds have strong binding affinity and stability. Notably, the free energy landscape plots where Rg and RMSD were used as coordinates showed strong thermodynamic stability due to the presence of stable conformers with global minima. This computational analysis provides a promising foundation for the further development of these compounds as potential antifungal drug candidates. The next step would be to conduct experimental validation through *in vitro* and *in vivo* studies to assess the compounds’ efficacy, pharmacokinetic properties, and safety profile. Additionally, these computational approaches can be extended to explore other potential drug targets of *C. albicans*, expanding the pipeline of novel antifungal therapies. The successful identification of these lead compounds through virtual screening highlights the power of *in silico* methods in accelerating the drug discovery process, especially for underexplored targets like Yck2. This study represents an important step towards addressing the pressing need for new antifungal drugs to combat the growing threat of invasive *Candida* infections.

## Data Availability

The original contributions presented in the study are included in the article/Supplementary Material, further inquiries can be directed to the corresponding authors.
