# Supplementary material for: Structural analogs of 2-(4-fluorophenyl)-6-methyl-3-(pyridin-4-yl)pyrazolo[1,5-a]pyridine for targeting Candida albicans non-essential stress kinase Yck2 through protein-ligand binding and dynamics analysis
Source: Front Chem. 2024 Aug 13;12:1430157. doi: 10.3389/fchem.2024.1430157 (PMC11347327; doi:10.3389/fchem.2024.1430157)
Supplement: Supplementary file 1 [file DataSheet1.docx]

Supplementary Material

Results

1. Virtual screening

Supplementary Table 1. Complete result of the virtual screening analysis of 589 chemical compounds.

| Compound | Model ID | Energy |
| --- | --- | --- |
| 102583821 | 1 | -11.2 |
| 12982634 | 1 | -10.9 |
| 102487860 | 1 | -10.9 |
| 86260205 | 1 | -10.8 |
| 102433329 | 1 | -10.8 |
| 14715923 | 1 | -10.8 |
| 59012357 | 1 | -10.8 |
| 102161513 | 1 | -10.7 |
| 139612412 | 1 | -10.6 |
| 10155254 | 1 | -10.6 |
| 389707 | 1 | -10.6 |
| 14715925 | 1 | -10.6 |
| 145956163 | 1 | -10.5 |
| 49765607 | 1 | -10.5 |
| 135007545 | 1 | -10.5 |
| 145957413 | 1 | -10.4 |
| 102316984 | 1 | -10.4 |
| 101912882 | 1 | -10.4 |
| 85705463 | 1 | -10.3 |
| 14065263 | 1 | -10.3 |
| 12902181 | 1 | -10.3 |
| 129835158 | 1 | -10.3 |
| 14715922 | 1 | -10.2 |
| 102287243 | 1 | -10.2 |
| 102385808 | 1 | -10.2 |
| 102316986 | 1 | -10.2 |
| 12747344 | 1 | -10.2 |
| 17990952 | 1 | -10.1 |
| 13447057 | 1 | -10.1 |
| 9836050 | 1 | -10.1 |
| 102129507 | 1 | -10.1 |
| 145958276 | 1 | -10.1 |
| 102076991 | 1 | -10.1 |
| 129839437 | 1 | -10.1 |
| 134942653 | 1 | -10 |
| 13013582 | 1 | -10 |
| 11694910 | 1 | -10 |
| 44308926 | 1 | -10 |
| 135003402 | 1 | -10 |
| 135540467 | 1 | -10 |
| 56697128 | 1 | -10 |
| 9922847 | 1 | -10 |
| 145949757 | 1 | -9.9 |
| 102287244 | 1 | -9.9 |
| 10707250 | 1 | -9.9 |
| 10016640 | 1 | -9.9 |
| 102316991 | 1 | -9.9 |
| 220298 | 1 | -9.9 |
| 102045751 | 1 | -9.9 |
| 95930149 | 1 | -9.8 |
| 102050809 | 1 | -9.8 |
| 89068 | 1 | -9.8 |
| 44308950 | 1 | -9.8 |
| 9857661 | 1 | -9.8 |
| 10614161 | 1 | -9.8 |
| 9814925 | 1 | -9.8 |
| 12230385 | 1 | -9.8 |
| 9857146 | 1 | -9.8 |
| 12533781 | 1 | -9.8 |
| 13573270 | 1 | -9.8 |
| 15534245 | 1 | -9.8 |
| 102150551 | 1 | -9.7 |
| 12038508 | 1 | -9.7 |
| 132496609 | 1 | -9.7 |
| 101510773 | 1 | -9.7 |
| 101515872 | 1 | -9.7 |
| 331057 | 1 | -9.7 |
| 85827014 | 1 | -9.7 |
| 102316985 | 1 | -9.7 |
| 90126424 | 1 | -9.7 |
| 11301180 | 1 | -9.7 |
| 155766 | 1 | -9.7 |
| 145955911 | 1 | -9.7 |
| 253613 | 1 | -9.6 |
| 71523920 | 1 | -9.6 |
| 1266746 | 1 | -9.6 |
| 13447055 | 1 | -9.6 |
| 145957118 | 1 | -9.6 |
| 102479990 | 1 | -9.6 |
| 102338082 | 1 | -9.6 |
| 14146272 | 1 | -9.6 |
| 102583818 | 1 | -9.6 |
| 122229034 | 1 | -9.6 |
| 102113604 | 1 | -9.6 |
| 389709 | 1 | -9.6 |
| 132538814 | 1 | -9.6 |
| 44341138 | 1 | -9.5 |
| 1211951 | 1 | -9.5 |
| 10017682 | 1 | -9.5 |
| 74536 | 1 | -9.5 |
| 9879081 | 1 | -9.5 |
| 71456547 | 1 | -9.5 |
| 102583820 | 1 | -9.5 |
| 71387049 | 1 | -9.5 |
| 11109504 | 1 | -9.5 |
| 13638864 | 1 | -9.5 |
| 135057537 | 1 | -9.5 |
| 14143414 | 1 | -9.5 |
| 16098034 | 1 | -9.5 |
| 101479662 | 1 | -9.5 |
| 18432770 | 1 | -9.5 |
| 10567234 | 1 | -9.5 |
| 11427473 | 1 | -9.5 |
| 11695604 | 1 | -9.5 |
| 122656 | 1 | -9.4 |
| 132539857 | 1 | -9.4 |
| 132581812 | 1 | -9.4 |
| 87638 | 1 | -9.4 |
| 11056061 | 1 | -9.4 |
| 145979203 | 1 | -9.4 |
| 71732324 | 1 | -9.4 |
| 137636377 | 1 | -9.4 |
| 253593 | 1 | -9.4 |
| 13382113 | 1 | -9.4 |
| 16072891 | 1 | -9.4 |
| 15827984 | 1 | -9.4 |
| 45113617 | 1 | -9.4 |
| 86260203 | 1 | -9.4 |
| 253597 | 1 | -9.3 |
| 13761322 | 1 | -9.3 |
| 133056913 | 1 | -9.3 |
| 12516009 | 1 | -9.3 |
| 122388827 | 1 | -9.3 |
| 101572721 | 1 | -9.3 |
| 101511165 | 1 | -9.3 |
| 11630626 | 1 | -9.3 |
| 102155314 | 1 | -9.3 |
| 101474482 | 1 | -9.3 |
| 12452197 | 1 | -9.3 |
| 85947490 | 1 | -9.3 |
| 11737689 | 1 | -9.3 |
| 137661 | 1 | -9.3 |
| 12247238 | 1 | -9.3 |
| 134842731 | 1 | -9.3 |
| 145952358 | 1 | -9.3 |
| 132539856 | 1 | -9.3 |
| 71325232 | 1 | -9.3 |
| 134928588 | 1 | -9.3 |
| 145953003 | 1 | -9.3 |
| 15992193 | 1 | -9.3 |
| 10643550 | 1 | -9.2 |
| 136811975 | 1 | -9.2 |
| 3084987 | 1 | -9.2 |
| 135026015 | 1 | -9.2 |
| 102433327 | 1 | -9.2 |
| 102265666 | 1 | -9.2 |
| 13468086 | 1 | -9.2 |
| 135051673 | 1 | -9.2 |
| 15430490 | 1 | -9.2 |
| 56958843 | 1 | -9.2 |
| 705644 | 1 | -9.2 |
| 753119 | 1 | -9.2 |
| 14528855 | 1 | -9.2 |
| 90666126 | 1 | -9.2 |
| 13761317 | 1 | -9.2 |
| 122229033 | 1 | -9.2 |
| 132539853 | 1 | -9.2 |
| 10449491 | 1 | -9.2 |
| 102155312 | 1 | -9.2 |
| 145953117 | 1 | -9.2 |
| 101083510 | 1 | -9.2 |
| 102193153 | 1 | -9.2 |
| 135057387 | 1 | -9.1 |
| 13866416 | 1 | -9.1 |
| 11289186 | 1 | -9.1 |
| 18342924 | 1 | -9.1 |
| 101500456 | 1 | -9.1 |
| 12672514 | 1 | -9.1 |
| 14247878 | 1 | -9.1 |
| 14715926 | 1 | -9.1 |
| 14547219 | 1 | -9.1 |
| 712785 | 1 | -9.1 |
| 102129506 | 1 | -9.1 |
| 145955947 | 1 | -9.1 |
| 10620766 | 1 | -9.1 |
| 102027940 | 1 | -9.1 |
| 145949860 | 1 | -9.1 |
| 86260201 | 1 | -9.1 |
| 146048274 | 1 | -9.1 |
| 10935149 | 1 | -9.1 |
| 102518975 | 1 | -9.1 |
| 101083509 | 1 | -9.1 |
| 11231922 | 1 | -9.1 |
| 56958844 | 1 | -9.1 |
| 11449070 | 1 | -9.1 |
| 101912879 | 1 | -9.1 |
| 135545386 | 1 | -9.1 |
| 409241 | 1 | -9.1 |
| 11291295 | 1 | -9.1 |
| 146048277 | 1 | -9.1 |
| 11551121 | 1 | -9 |
| 139204561 | 1 | -9 |
| 145959344 | 1 | -9 |
| 102385815 | 1 | -9 |
| 102155315 | 1 | -9 |
| 134841049 | 1 | -9 |
| 139225462 | 1 | -9 |
| 164683953 | 1 | -9 |
| 85684442 | 1 | -9 |
| 13522537 | 1 | -9 |
| 57412727 | 1 | -9 |
| 49765606 | 1 | -9 |
| 11139857 | 1 | -9 |
| 47003438 | 1 | -9 |
| 11243189 | 1 | -9 |
| 101519051 | 1 | -9 |
| 15446602 | 1 | -9 |
| 102161514 | 1 | -9 |
| 15456185 | 1 | -9 |
| 1207762 | 1 | -9 |
| 3816730 | 1 | -9 |
| 12618643 | 1 | -9 |
| 1547691 | 1 | -9 |
| 122379041 | 1 | -9 |
| 6613391 | 1 | -9 |
| 102316983 | 1 | -9 |
| 12403204 | 1 | -9 |
| 469038 | 1 | -9 |
| 101786532 | 1 | -9 |
| 15193132 | 1 | -9 |
| 95930101 | 1 | -9 |
| 13358898 | 1 | -8.9 |
| 14715924 | 1 | -8.9 |
| 102287242 | 1 | -8.9 |
| 146048278 | 1 | -8.9 |
| 12505674 | 1 | -8.9 |
| 13027057 | 1 | -8.9 |
| 135540463 | 1 | -8.9 |
| 85726985 | 1 | -8.9 |
| 139242213 | 1 | -8.9 |
| 14547218 | 1 | -8.9 |
| 44395394 | 1 | -8.9 |
| 11482926 | 1 | -8.9 |
| 47003436 | 1 | -8.9 |
| 11186946 | 1 | -8.9 |
| 2916540 | 1 | -8.9 |
| 101446367 | 1 | -8.9 |
| 101912890 | 1 | -8.9 |
| 102479989 | 1 | -8.9 |
| 269243 | 1 | -8.9 |
| 129839534 | 1 | -8.9 |
| 11267034 | 1 | -8.9 |
| 12859431 | 1 | -8.9 |
| 11426567 | 1 | -8.9 |
| 15456184 | 1 | -8.9 |
| 3872336 | 1 | -8.9 |
| 101911602 | 1 | -8.9 |
| 102433326 | 1 | -8.8 |
| 4080631 | 1 | -8.8 |
| 3491524 | 1 | -8.8 |
| 628994 | 1 | -8.8 |
| 86122145 | 1 | -8.8 |
| 101911603 | 1 | -8.8 |
| 12339686 | 1 | -8.8 |
| 10517320 | 1 | -8.8 |
| 45488169 | 1 | -8.8 |
| 71456545 | 1 | -8.8 |
| 129738777 | 1 | -8.8 |
| 11290472 | 1 | -8.8 |
| 155540824 | 1 | -8.8 |
| 129839290 | 1 | -8.8 |
| 145959544 | 1 | -8.8 |
| 132966487 | 1 | -8.8 |
| 102027941 | 1 | -8.8 |
| 101503130 | 1 | -8.8 |
| 59012351 | 1 | -8.8 |
| 12859430 | 1 | -8.8 |
| 145990401 | 1 | -8.8 |
| 86260202 | 1 | -8.8 |
| 10779709 | 1 | -8.8 |
| 16114731 | 1 | -8.8 |
| 122379048 | 1 | -8.8 |
| 13278580 | 1 | -8.8 |
| 12339685 | 1 | -8.7 |
| 102076987 | 1 | -8.7 |
| 12536706 | 1 | -8.7 |
| 12958549 | 1 | -8.7 |
| 6425397 | 1 | -8.7 |
| 58880595 | 1 | -8.7 |
| 86181039 | 1 | -8.7 |
| 5039735 | 1 | -8.7 |
| 146048273 | 1 | -8.7 |
| 12649526 | 1 | -8.7 |
| 12074383 | 1 | -8.7 |
| 16114735 | 1 | -8.7 |
| 10788138 | 1 | -8.7 |
| 44341178 | 1 | -8.7 |
| 134957996 | 1 | -8.7 |
| 10063955 | 1 | -8.7 |
| 86260222 | 1 | -8.7 |
| 16000126 | 1 | -8.7 |
| 11781932 | 1 | -8.7 |
| 101083511 | 1 | -8.7 |
| 102171716 | 1 | -8.7 |
| 11289743 | 1 | -8.7 |
| 101455491 | 1 | -8.7 |
| 416748 | 1 | -8.7 |
| 122220285 | 1 | -8.7 |
| 9884218 | 1 | -8.7 |
| 13280242 | 1 | -8.7 |
| 13230684 | 1 | -8.7 |
| 14065268 | 1 | -8.7 |
| 21988507 | 1 | -8.7 |
| 49852168 | 1 | -8.7 |
| 162400231 | 1 | -8.7 |
| 101911604 | 1 | -8.7 |
| 1484085 | 1 | -8.7 |
| 136109023 | 1 | -8.7 |
| 19353993 | 1 | -8.7 |
| 102583816 | 1 | -8.7 |
| 9838979 | 1 | -8.6 |
| 25242160 | 1 | -8.6 |
| 137659530 | 1 | -8.6 |
| 135499794 | 1 | -8.6 |
| 102111474 | 1 | -8.6 |
| 854671 | 1 | -8.6 |
| 11551202 | 1 | -8.6 |
| 10542335 | 1 | -8.6 |
| 71512586 | 1 | -8.6 |
| 154717418 | 1 | -8.6 |
| 12241720 | 1 | -8.6 |
| 15470495 | 1 | -8.6 |
| 45104351 | 1 | -8.6 |
| 16114732 | 1 | -8.6 |
| 23649580 | 1 | -8.6 |
| 101511163 | 1 | -8.6 |
| 10891931 | 1 | -8.6 |
| 69096714 | 1 | -8.6 |
| 11461667 | 1 | -8.6 |
| 10892277 | 1 | -8.6 |
| 10266983 | 1 | -8.6 |
| 102171713 | 1 | -8.6 |
| 134151653 | 1 | -8.6 |
| 10756761 | 1 | -8.6 |
| 129034 | 1 | -8.6 |
| 21495911 | 1 | -8.6 |
| 132513341 | 1 | -8.6 |
| 10707332 | 1 | -8.5 |
| 102579258 | 1 | -8.5 |
| 318543 | 1 | -8.5 |
| 101912880 | 1 | -8.5 |
| 83430969 | 1 | -8.5 |
| 13013583 | 1 | -8.5 |
| 15577976 | 1 | -8.5 |
| 135409746 | 1 | -8.5 |
| 44340852 | 1 | -8.5 |
| 873105 | 1 | -8.5 |
| 100929958 | 1 | -8.5 |
| 12649525 | 1 | -8.5 |
| 739096 | 1 | -8.5 |
| 422988 | 1 | -8.5 |
| 3241417 | 1 | -8.5 |
| 9879492 | 1 | -8.5 |
| 11426096 | 1 | -8.5 |
| 141447307 | 1 | -8.5 |
| 57403738 | 1 | -8.5 |
| 722214 | 1 | -8.5 |
| 9836051 | 1 | -8.5 |
| 1486686 | 1 | -8.5 |
| 13284085 | 1 | -8.5 |
| 60136332 | 1 | -8.5 |
| 58603311 | 1 | -8.5 |
| 11391610 | 1 | -8.4 |
| 13625886 | 1 | -8.4 |
| 132850738 | 1 | -8.4 |
| 102245891 | 1 | -8.4 |
| 142723511 | 1 | -8.4 |
| 24850011 | 1 | -8.4 |
| 10853235 | 1 | -8.4 |
| 101877142 | 1 | -8.4 |
| 13612544 | 1 | -8.4 |
| 15500896 | 1 | -8.4 |
| 132513340 | 1 | -8.4 |
| 122385730 | 1 | -8.4 |
| 9945696 | 1 | -8.4 |
| 13173990 | 1 | -8.4 |
| 71385061 | 1 | -8.4 |
| 59359524 | 1 | -8.4 |
| 3525174 | 1 | -8.4 |
| 71451213 | 1 | -8.4 |
| 71454774 | 1 | -8.4 |
| 13126838 | 1 | -8.4 |
| 13899986 | 1 | -8.4 |
| 102415657 | 1 | -8.4 |
| 24873257 | 1 | -8.4 |
| 71284143 | 1 | -8.4 |
| 134913532 | 1 | -8.4 |
| 1274323 | 1 | -8.4 |
| 1209050 | 1 | -8.4 |
| 747503 | 1 | -8.4 |
| 10131654 | 1 | -8.4 |
| 129795579 | 1 | -8.4 |
| 9923028 | 1 | -8.4 |
| 19022712 | 1 | -8.4 |
| 13095079 | 1 | -8.4 |
| 101511167 | 1 | -8.3 |
| 633887 | 1 | -8.3 |
| 298651 | 1 | -8.3 |
| 9901062 | 1 | -8.3 |
| 162403084 | 1 | -8.3 |
| 752153 | 1 | -8.3 |
| 15618707 | 1 | -8.3 |
| 10912627 | 1 | -8.3 |
| 12964122 | 1 | -8.3 |
| 10470311 | 1 | -8.3 |
| 2405153 | 1 | -8.3 |
| 162403086 | 1 | -8.3 |
| 137635554 | 1 | -8.3 |
| 45488168 | 1 | -8.3 |
| 71399595 | 1 | -8.3 |
| 15313367 | 1 | -8.3 |
| 9944397 | 1 | -8.3 |
| 162419015 | 1 | -8.3 |
| 11694814 | 1 | -8.3 |
| 11208609 | 1 | -8.3 |
| 11208888 | 1 | -8.3 |
| 71460240 | 1 | -8.3 |
| 10827088 | 1 | -8.3 |
| 20826170 | 1 | -8.3 |
| 12224155 | 1 | -8.2 |
| 3541772 | 1 | -8.2 |
| 122388825 | 1 | -8.2 |
| 57394999 | 1 | -8.2 |
| 58787449 | 1 | -8.2 |
| 45487937 | 1 | -8.2 |
| 102113606 | 1 | -8.2 |
| 136609929 | 1 | -8.2 |
| 15964164 | 1 | -8.2 |
| 13835827 | 1 | -8.2 |
| 12142897 | 1 | -8.2 |
| 624943 | 1 | -8.2 |
| 9858407 | 1 | -8.2 |
| 21573688 | 1 | -8.2 |
| 134853816 | 1 | -8.2 |
| 162403088 | 1 | -8.2 |
| 10850716 | 1 | -8.2 |
| 15577975 | 1 | -8.2 |
| 71456544 | 1 | -8.2 |
| 135818779 | 1 | -8.2 |
| 11427498 | 1 | -8.2 |
| 102520516 | 1 | -8.1 |
| 804290 | 1 | -8.1 |
| 15993465 | 1 | -8.1 |
| 89876237 | 1 | -8.1 |
| 13040235 | 1 | -8.1 |
| 15827985 | 1 | -8.1 |
| 83426989 | 1 | -8.1 |
| 122379043 | 1 | -8.1 |
| 122196041 | 1 | -8.1 |
| 102113605 | 1 | -8.1 |
| 5287525 | 1 | -8.1 |
| 389705 | 1 | -8.1 |
| 102113603 | 1 | -8.1 |
| 57145268 | 1 | -8.1 |
| 15263128 | 1 | -8.1 |
| 11407517 | 1 | -8.1 |
| 21392788 | 1 | -8.1 |
| 3541770 | 1 | -8 |
| 19354014 | 1 | -8 |
| 102579259 | 1 | -8 |
| 71512804 | 1 | -8 |
| 13526428 | 1 | -8 |
| 469040 | 1 | -8 |
| 102133826 | 1 | -8 |
| 13526455 | 1 | -8 |
| 90666124 | 1 | -8 |
| 3541771 | 1 | -8 |
| 12601282 | 1 | -8 |
| 15827986 | 1 | -8 |
| 44151317 | 1 | -8 |
| 10851778 | 1 | -8 |
| 132501674 | 1 | -8 |
| 14966167 | 1 | -8 |
| 57391517 | 1 | -7.9 |
| 10809449 | 1 | -7.9 |
| 102316990 | 1 | -7.9 |
| 2403371 | 1 | -7.9 |
| 101511164 | 1 | -7.9 |
| 13164504 | 1 | -7.9 |
| 134957994 | 1 | -7.9 |
| 11449071 | 1 | -7.9 |
| 101892551 | 1 | -7.9 |
| 9879605 | 1 | -7.9 |
| 122196049 | 1 | -7.9 |
| 44290409 | 1 | -7.9 |
| 13027064 | 1 | -7.9 |
| 102497316 | 1 | -7.9 |
| 14642213 | 1 | -7.9 |
| 10517468 | 1 | -7.9 |
| 139207570 | 1 | -7.9 |
| 44606944 | 1 | -7.8 |
| 19022684 | 1 | -7.8 |
| 101116863 | 1 | -7.8 |
| 102235992 | 1 | -7.8 |
| 712787 | 1 | -7.8 |
| 12689666 | 1 | -7.8 |
| 101800403 | 1 | -7.8 |
| 10016305 | 1 | -7.8 |
| 290983 | 1 | -7.8 |
| 10084790 | 1 | -7.8 |
| 752154 | 1 | -7.7 |
| 138964275 | 1 | -7.7 |
| 146048228 | 1 | -7.7 |
| 101792854 | 1 | -7.7 |
| 16069621 | 1 | -7.7 |
| 755814 | 1 | -7.7 |
| 101447812 | 1 | -7.7 |
| 3106812 | 1 | -7.7 |
| 101134706 | 1 | -7.7 |
| 44603688 | 1 | -7.6 |
| 16114854 | 1 | -7.6 |
| 4164643 | 1 | -7.6 |
| 874735 | 1 | -7.6 |
| 83426991 | 1 | -7.6 |
| 12283178 | 1 | -7.6 |
| 69715146 | 1 | -7.6 |
| 10540421 | 1 | -7.6 |
| 12188978 | 1 | -7.6 |
| 13761327 | 1 | -7.5 |
| 102517223 | 1 | -7.5 |
| 101510774 | 1 | -7.5 |
| 102583819 | 1 | -7.5 |
| 12689668 | 1 | -7.5 |
| 68412717 | 1 | -7.5 |
| 132489470 | 1 | -7.5 |
| 154714478 | 1 | -7.5 |
| 132581810 | 1 | -7.5 |
| 71428276 | 1 | -7.5 |
| 10108290 | 1 | -7.5 |
| 15430489 | 1 | -7.5 |
| 139095138 | 1 | -7.5 |
| 129725 | 1 | -7.4 |
| 59866501 | 1 | -7.4 |
| 11199396 | 1 | -7.4 |
| 44407637 | 1 | -7.4 |
| 10690316 | 1 | -7.4 |
| 137646042 | 1 | -7.4 |
| 132513339 | 1 | -7.4 |
| 10730073 | 1 | -7.4 |
| 13143688 | 1 | -7.4 |
| 69714556 | 1 | -7.4 |
| 139072496 | 1 | -7.4 |
| 44340831 | 1 | -7.4 |
| 12291590 | 1 | -7.3 |
| 44589676 | 1 | -7.3 |
| 101877143 | 1 | -7.3 |
| 135007544 | 1 | -7.3 |
| 129787928 | 1 | -7.3 |
| 59867354 | 1 | -7.3 |
| 102294274 | 1 | -7.2 |
| 162665760 | 1 | -7.2 |
| 738804 | 1 | -7.2 |
| 19353962 | 1 | -7.2 |
| 102027942 | 1 | -7.2 |
| 44341122 | 1 | -7.2 |
| 44142571 | 1 | -7.2 |
| 10688435 | 1 | -7.1 |
| 11810212 | 1 | -7.1 |
| 268041 | 1 | -7.1 |
| 91169918 | 1 | -7.1 |
| 102179226 | 1 | -7 |
| 129798016 | 1 | -7 |
| 102310799 | 1 | -7 |
| 102577409 | 1 | -7 |
| 13466014 | 1 | -7 |
| 71726190 | 1 | -7 |
| 87264666 | 1 | -7 |
| 102266519 | 1 | -6.9 |
| 4064491 | 1 | -6.9 |
| 13526382 | 1 | -6.9 |
| 3498086 | 1 | -6.9 |
| 12601281 | 1 | -6.9 |
| 469039 | 1 | -6.9 |
| 11795989 | 1 | -6.9 |
| 13027063 | 1 | -6.8 |
| 628540 | 1 | -6.8 |
| 15155516 | 1 | -6.8 |
| 3966436 | 1 | -6.8 |
| 89876180 | 1 | -6.8 |
| 10760956 | 1 | -6.8 |
| 849795 | 1 | -6.7 |
| 13526404 | 1 | -6.6 |
| 46228214 | 1 | -6.6 |
| 13526390 | 1 | -6.6 |
| 4031572 | 1 | -6.5 |
| 767558 | 1 | -6.5 |
| 5228 | 1 | -6.2 |
| 131747632 | 1 | -6.2 |
| 11290175 | 1 | -6.1 |


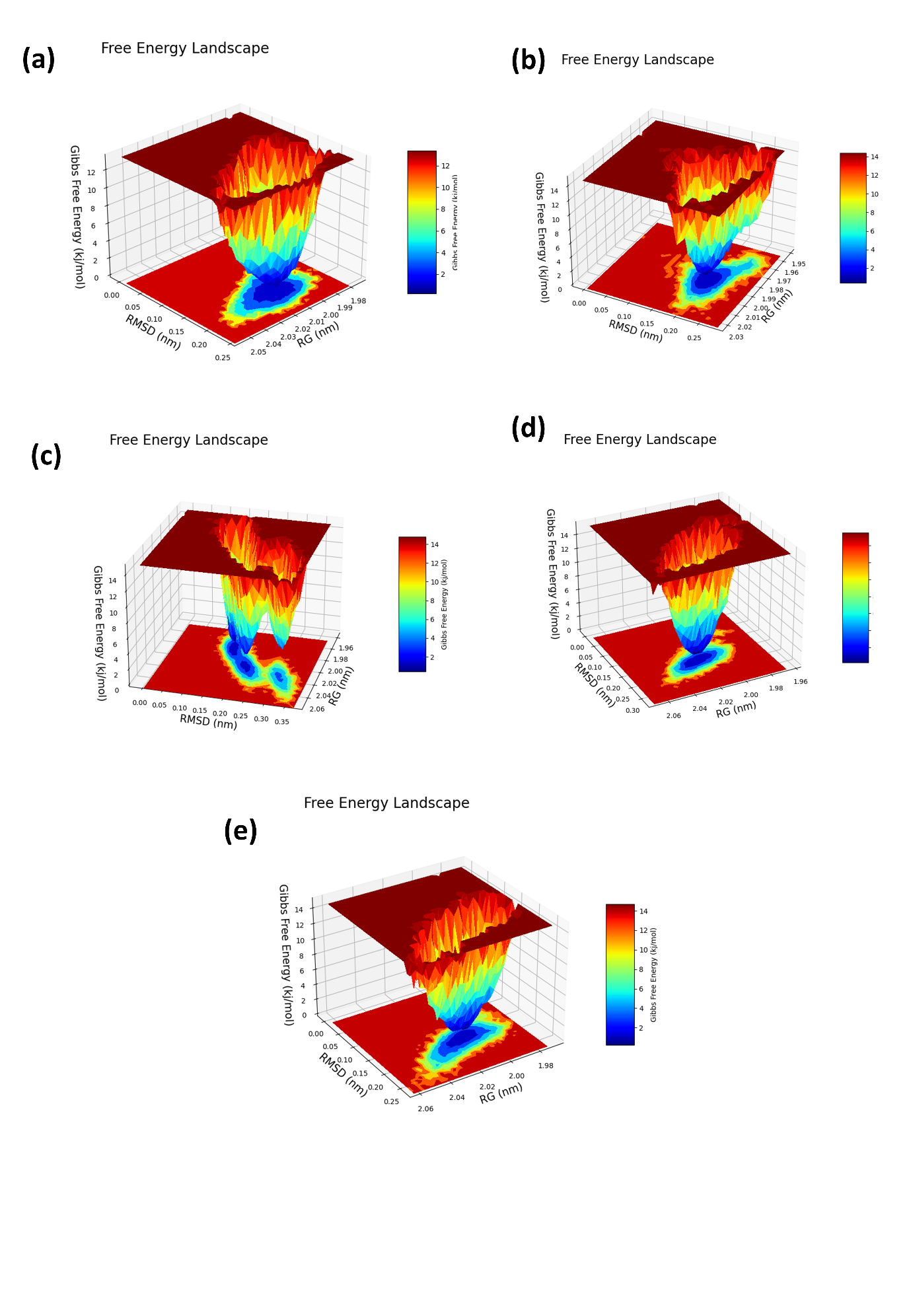
Supplementary Figure 1. 3D Fel plot of of the following complexes (a) Yck2-102583821 (b) Yck2-12982634 (c) Yck2-102487860 (d) Yck2-86260205 and (e) Yck2-Q0J (reference)
